# Disease of the Cavities of Molar Teeth in Horses

**Published:** 1858-01

**Authors:** 


*Disease of the Cavities of Molar Teeth in Horses.*—In the September number of *L'Art Dentaire,* we find an account of a peculiar affection of the teeth of horses, taken from the *Annales de Medicine Veterinaire.* This consists of a loosening and deviation of the molar teeth of the lower jaws so that fodder gets between them during mastication. By repeated compression the fodder is forced into the alveolar cavity and occasions violent pain to the animal, which is thus prevented from masticating properly. This trouble is not necessarily attended by disease of the teeth, and always occurs on both sides at the same time. It is easily recognized by the attentive veterinary surgeon, who, observing a painful mastication accompanied by an emaciation of the animal, never fails to examine the mouth, then he will find food compressed between two teeth and exhaling a very disagreeable odor; on removing the food with the fingers, an interval will be found between the teeth. According to the observations of Müller, this interval is almost always formed at the side of the fourth molar, between it and the third, or between it and the fifth.

He attributes the cause of this disease to a vicious conformation of the maxillary bone, and considers it incurable. He advises the removal of the deviating tooth, and warns the operator to take care that the corresponding tooth of the other jaw does not bury itself too far forward in the artificial tooth, but he adds, that, though at first after this operation, the animals eat a little better, this amelioration was always of short duration, so that in the end he was forced to give up the horses which were sent to him affected with this malady.

